# Metformin paradoxically worsens insulin resistance in SHORT syndrome

**DOI:** 10.1186/s13098-019-0477-z

**Published:** 2019-10-01

**Authors:** Krzysztof C. Lewandowski, Katarzyna Dąbrowska, Maria Brzozowska, Joanna Kawalec, Andrzej Lewiński

**Affiliations:** 1 Department of Endocrinology and Metabolic Diseases, Medical University of Lodz, Lodz, Poland; 2 Department of Endocrinology and Metabolic Diseases, Polish Mother’s Memorial Hospital-Research Institute, Lodz, Poland

**Keywords:** SHORT syndrome, Metformin, Insulin resistance

## Abstract

**Background:**

SHORT syndrome is an autosomal dominant condition associated severe insulin resistance (IR) and lipoatrophy due to post-receptor defect in insulin signaling involving phosphoinositide-3-kinase regulatory subunit 1 (PIK3R1), where no clear treatment guidelines are available.

**Methods:**

We attempted to test the efficacy metformin in a female patient with SHORT syndrome by measuring glucose and insulin during an extended Oral Glucose Tolerance Test (OGTT) in a 21-year old patient (BMI 17.5 kg/m^2^), who presented for endocrine assessment with a history of amenorrhoea.

**Results:**

She had lipid concentrations within the reference range, normal thyroid function tests, prolactin, gonadotropins, estradiol and androgens with Free Androgen Index 4.52. Extended Oral Glucose Tolerance Test was performed and showed severe IR. She was then started on metformin 850 mg twice a day, and had repeated OGTT. This showed dramatic worsening of glucose tolerance (e.g. glucose 96 mg/dl versus 187 mg/dl and 68 mg/dl versus 204 mg/dl at 120 and 150 min of OGTT, respectively). This was accompanied by a massive increase of already high insulin concentrations (e.g. from 488.6 to > 1000 µIU/ml, and from 246.8 to > 1000 µIU/ml at 120 and 150 min of OGTT, respectively). Insulin concentrations remained above upper assay detection limit also at 180 min of OGTT on metformin treatment (> 1000 µIU/ml versus 100.6 µIU/ml without metformin).

**Conclusions:**

Metformin treatment may paradoxically lead to deterioration of insulin resistance and to development of glucose intolerance in SHORT syndrome. Hence, metformin treatment might be potentially harmful in these patients. Though, the precise cause of such profound and paradoxical worsening of glucose tolerance post metformin remains unknown, SHORT syndrome might prove to be an interesting model to study the mechanism(s) of metformin action.

## Background

SHORT syndrome is an autosomal dominant condition associated with severe insulin resistance and early onset of type 2 diabetes in the absence of obesity. The acronym stands for Short stature, Hyperextensibility, Ocular depression, Rieger anomaly (i.e. developmental anomaly of the iris also called Axenfeld-Rieger anomaly), Teething delay. Phenotypic presentation [1, 2] involves characteristic triangular face with deeply set eyes, prominent forehead and thin nasal alae, relatively short stature and often a history of an intrauterine growth restriction. SHORT syndrome is also characterized by partial lipodystrophy and severe insulin resistance (IR), leading to an early onset of type 2 diabetes, due to immediate post-receptor defect in insulin signaling (phosphoinositide-3-kinase regulatory subunit 1-PIK3R1) [2, 3]. According to the most comprehensive overview of SHORT syndrome [4], the cardinal feature of this condition include facial gestalt as well as growth and endocrine abnormalities (including clinical presentation of polycystic ovary syndrome).

## Case description

We describe a case of 21-year old female patient who presented for an endocrine assessment in our Department with a history of amenorrhoea. SHORT syndrome was diagnosed at the age of 16, and simultaneously this diagnosis was also established in her father with insulin-treated type 2 diabetes and younger brother, then aged 13. In all three family members the diagnosis was made at the Department of Dentistry at the Medical University of Lodz (Poland), where delayed dentition and enamel hypoplasia associated with facial gestalt (triangular face with deep set eyes and prominent forehead in both children and their father) i.e. typical features associated with the SHORT syndrome as described by Avila et al. [4], prompted referral to the Department of Genetics. Genetic testing (MEDGEN Warsaw) confirmed mutation in PIK3R1 locus (NM_181523.2:c.1945C > T)—copy of original report available on request. As described by Chudasama et al. [1] and Innes and Dyment [2] the described nucleotide change (c.1945C > T, with predicted p.Arg649Trp protein change) constitutes the most recurrent pathogenic variant of the SHORT syndrome, described in 10 out of 16 SHORT families [2].

## Investigation results

Her height was 158 cm, weight 43.7 kg, BMI 17.5 kg/m^2^. She had severe insulin resistance, both based on fasting glucose and insulin concentrations (HOMA-IR) and on glucose and insulin concentrations during OGTT [Insulin Resistance (Belfiore) Index] despite clinical features of lipoatrophy. Pelvic ultrasound (performed as an outpatient) showed polycystic ovaries. She had no spontaneous menstrual cycles, however, she had period induced by progestogen administration (Dydrogesterone—Duphaston^®^) suggestive of an adequate oestrogenisation.

Results of her biochemical and hormonal tests are presented in Table 1.

**Table 1:** Biochemical and hormonal results of 21 year old women with SHORT syndrome

Parameter	Value	Reference range
Fat mass (kg)	6.2	10–18.4
Body fat percentage (%)	14.3	21––32.9
Total cholesterol (mg/dl)	156	< 200
LDL cholesterol (mg/dl)	74.0	< 100
HDL cholesterol (mg/dl)	60.0	> 40
Triglycerides (mg/dl)	99.0	< 150
TSH (µIU/ml)	1.49	0.27–4.2
Free T4 (ng/dl)	1.12	0.93–1.7
Prolactin (ng/ml)	11.2	4.79–23.3
Total testosterone (ng/ml)	0.45	0.084–0.481
SHBG (nmol/l)	34.7	18–144
DHEAS (µg/dl)	147.4	148–407
Calcium (mmol/l)	2.50	2.10–2.55
PTH (pg/ml)	28.31	15–65
Vitamin D (ng/ml)	11.0	> 20
HOMA-IR [5]	7.64	No clear ref. range: 90th percentile usually around 3.46–3.8 [7]
Insulin resistance (Belfiore) Index [6]	1.85	No clear ref. range, values > 1.27 suggested as cut-off [8]

**Fig. 1:**
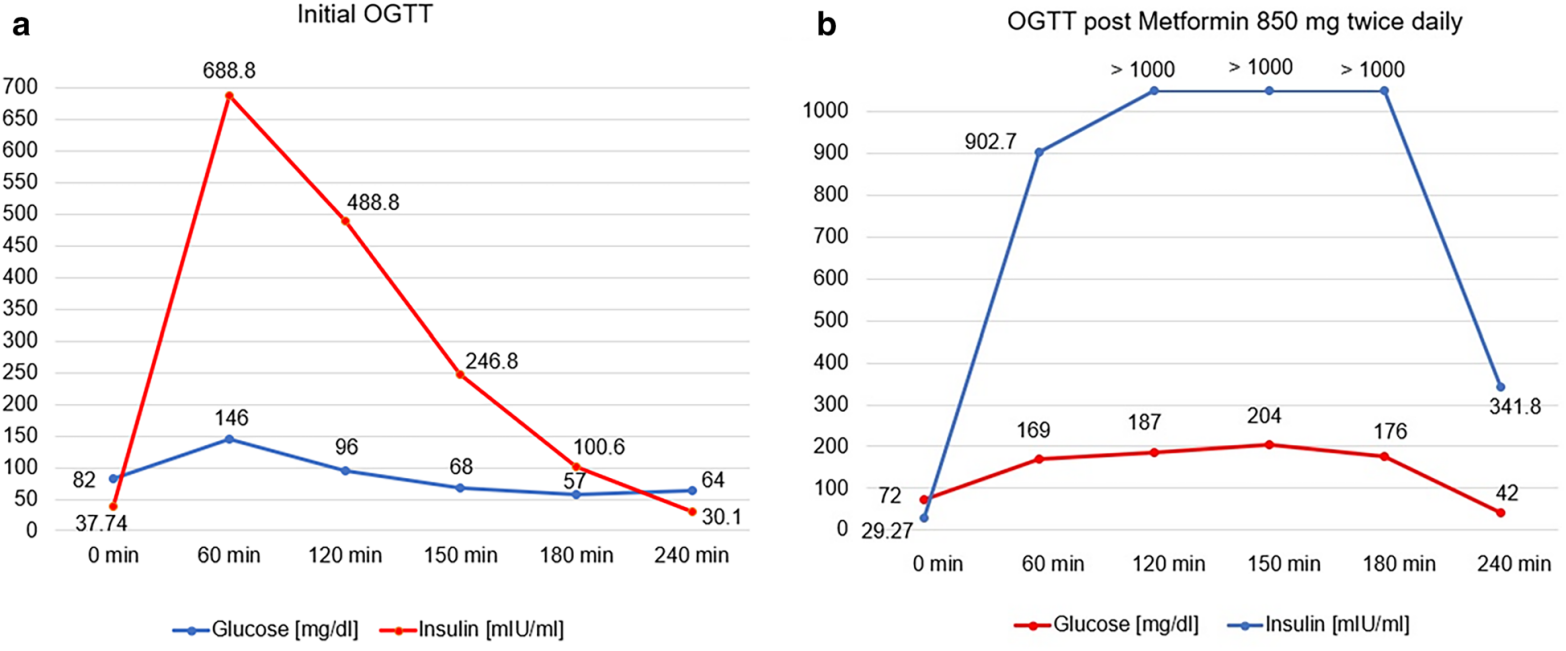
Results of 75 g OGTT before (**a**) and after (**b**) Metformin treatment in 21 year old women with SHORT syndrome

She also reported symptoms suggestive of delayed postprandial hypoglycaemia, so an extended 75 g OGTT was performed. This indeed showed severe IR and reactive hypoglycaemia (Fig. 1a).

In view of such severe insulin resistance as well as period problems, treatment with metformin (850 mg twice daily) was instigated. As we intended to check the effects of this approach, an extended 75 g OGTT was performed on metformin 4 days later. This showed dramatic and paradoxical worsening of insulin tolerance with insulin concentrations above the upper assay detection limit (Fig. 1b). Metformin treatment was discontinued. She was discharged home on Dydrogesterone and vitamin D supplementation. We planned to perform investigations on other family members, and particularly on her younger brother, but despite several reminders they failed to attend clinic appointments as well as declined admission to the hospital.

## Discussion

Our patient demonstrated typical features of the SHORT syndrome, with severe insulin resistance and lipoatrophy in the absence of dyslipidaemia, as described before [1]. In women of reproductive age (like in our case), presentation of the SHORT syndrome may also mimic classical polycystic ovary syndrome [4]. The precise cause of such profound and paradoxical worsening of glucose tolerance post metformin remains unknown. The mechanism of action of metformin is complex, but it involves several post-receptor steps in insulin signaling, such as activation of AMP kinase, effects on mitochondrial ATP production, thus resulting in an increase of cytoplasmic ADP:ATP and AMP:ATP ratios, as well as other effects including composition of gut microbiota [9]. In contrast, mutation in insulin signalling causing the SHORT syndrome (PI3K) affects very immediate post-receptor steps of insulin signalling [3]. Recent evidence suggests that metformin might also exert an anti-cancer effects [10], that may also involve at least some partial blockade of PI3K/MAPK signaling pathway, that is also implicated in cell growth [11]. If metformin indeed partially inhibits PI3K, then we speculate that further inhibition of this already mutated enzyme might have prevented any beneficial effects of metformin, as these generally affect further i.e. down-stream steps of insulin signaling. In such circumstances, further inhibition of PIK3R1 could have worsened insulin resistance in the setting of metformin treatment. This hypothesis, however, requires further study.

Nevertheless, regardless of a precise mechanism, we observed a severe and an unequivocal worsening of insulin resistance on metformin treatment in a patient with a classical SHORT syndrome mutation (NM_181523.2: c.1945C > T). This points to the possibility that SHORT syndrome model might be useful for further research into the insight of metformin action.

There are also clinical implications of our case. Doctors very rarely perform glucose tolerance tests on metformin, as this agent is usually stopped prior to testing, in order to avoid possible false negative results. Furthermore, in most cases OGTT is performed without concomitant insulin measurements. In this case we performed the second OGTT on metformin in order to assess the extent of improvement of insulin resistance in this case of rare genetically-determined disease associated with severe insulin resistance. Paradoxically we observed worsening of glucose tolerance with a massive release of insulin above the assay detection limit. If the second Oral Glucose Tolerance Test (i.e. on metformin treatment) had not been performed, then subsequent worsening of glucose tolerance, or even early development of type 2 diabetes would have been most likely attributed to beta-cell exhaustion due to genetically-determined insulin resistance, rather to superimposed effects of metformin treatment, that would have been missed. In case of the SHORT syndrome, most available literature data [1, 2, 4, 12, 13] pertain to either genetic or clinical characteristics of disease with hardly any data on treatment modalities and outcome. For instance, in an extensive genetic paper by Huang-Doran et al. [14], it is mentioned that “treatment with metformin and combined cyproterone acetate/ethinylestradiol preparation was begun”, however, without any data on treatment outcome. Verge et al. [15] report apparently successful treatment with metformin in a 14-year boy with SHORT syndrome, but this situation is difficult to compare with our case as development of type 2 diabetes was thought to be induced by growth hormone treatment (known to worsen insulin resistance), and diabetes subsided few months after discontinuation of growth hormone.

## Conclusions

The case of our patient therefore constitutes a caution for clinicians, who treat insulin-resistant states, where metformin is usually a drug of the first choice. SHORT syndrome might represent an exception from this rule. Indeed, at least in some of these patients metformin treatment might worsen, rather than improve, glucose tolerance, and paradoxically accelerate an onset of type 2 diabetes. In view of these findings we postulate that SHORT syndrome might be a model to test yet unknown aspects of metformin actions.

## Data Availability

A copy of the original discharge summary (in an anonymous form) may be available on Editors’s request. As the study comprises a case report, then no formal database has been created.

## Abbreviations

IR: insulin resistance
PIK3R1: phosphoinositide-3-kinase regulatory subunit 1
OGTT: Oral Glucose Tolerance Test
HOMA-IR: Homeostatic Model Assessment of Insulin Resistance
TSH: thyroid-stimulating hormone
T4: thyroxine
SHGB: sex hormone binding globulin
DHEAS: dehydroepiandrosterone sulphate
PTH: parathyroid hormone
ATP: adenosine triphosphate
AMP: adenosine monophosphate
